# Quality of life in the course of a one-year use of an advanced hybrid closed-loop system in adults with type 1 diabetes previously naïve to advanced diabetes technology

**DOI:** 10.3389/fendo.2023.1210756

**Published:** 2023-08-15

**Authors:** Katarzyna Cyranka, Bartłomiej Matejko, Anna Juza, Beata Kieć-Wilk, Ohad Cohen, Maciej T. Malecki, Tomasz Klupa

**Affiliations:** ^1^ Department of Metabolic Diseases, Jagiellonian University Medical College, Krakow, Poland; ^2^ University Hospital in Krakow, Krakow, Poland; ^3^ Department of Psychiatry, Jagiellonian University Medical College, Krakow, Poland; ^4^ Clinical Provincial Hospital of Frederic Chopin No. 1 in Rzeszów, Rzeszów, Poland; ^5^ College of Medical Sciences, University of Rzeszów, Rzeszów, Poland; ^6^ Medtronic, Tolochenaz, Switzerland

**Keywords:** quality of life, advanced hybrid closed-loop system, diabetes type 1, self-efficacy, anxiety

## Abstract

**Aim:**

To evaluate the effect of a one-year use of an advanced hybrid closed-loop (AHCL) system on the quality of life, level of anxiety, and level of self-efficacy in adults with type 1 diabetes (T1D) previously treated with multiple daily injections (MDI) and naïve to advanced diabetes technology

**Methods:**

A total of 18 participants of a previously published 3-month randomized trial (10 men, 8 women; age 40.9 ± 7.6 years) who were switched directly from MDI/BMG to AHCL completed 12 months of MiniMed 780G™system use (a 3-month randomized trial followed by a 9-month follow-up phase). At month 6 of the study, patients were switched from the sensor GS3 (Continuous Glucose Monitoring) system, powered by Guardian™ Sensor 3) to GS4. Quality of life was assessed using the Polish validated version of the ‘QoL-Q Diabetes’ questionnaire. The level of anxiety was evaluated with the use of the State-Trait Anxiety Inventory (STAI). Self-efficacy was assessed with the General Self-Efficacy Scale (GSES). Results were obtained at baseline and at the end of the study.

**Results:**

Significant increase in QoL was reported in the global score (p=0.02, Cohen d=0.61) and in as many as 11 out of 23 analyzed areas of life: being physically active (p=0.02, Cohen d = 0.71); feeling well (p<.01, Cohen d = 0.73); feeling in control of my body (p<.01, Cohen d = 0.72); looking good (p<.01, Cohen d = 1.07); working (p<.01, Cohen d = 1.12); sleeping (p=0.01, Cohen d = 0.66); eating as I would like (p<.01, Cohen d = 0.79); looking after or being useful to others (p= 0.02, Cohen d = 0.65); being active with pets/animals (p<.01, Cohen d = 0.95); being spontaneous (p=0.02, Cohen d = 0.67); and doing “normal” things (p=0.02, Cohen d = 0.67). Both state (p=0.04, Cohen d = 0.56) and trait (p=0.02, Cohen d = 0.60) anxiety decreased while the general self-efficacy increased (p=0.03, Cohen d = 0.76). No participant stopped the use of the pump.

**Conclusion:**

Adult patients with T1D previously treated with MDI and naïve to modern technologies experienced significant improvement in their psychological well-being after transitioning to the AHCL system after 12 months of treatment.

## Introduction

Quality of life (QoL) is a crucial concept in the assessment of individual functioning, general health, and well-being (1). Health-related quality of life (HRQoL) indicates the impact of chronic disease on the health status of the patient (2, 3). HRQoL assessment allows identifying those aspects of patient functioning that require intervention (4). Evaluation of QoL should be a standard procedure in the assessment of the effectiveness of any newly applied treatment, especially in diabetes - a disease that requires a lot of engagement of the patients in all aspects of their life (5). The use of Continuous Subcutaneous Insulin Infusion (CSII) and Continuous Glucose Monitoring (CGM) used separately are associated with an improvement in glycemic control. Modern technological advances have integrated CSII with CGM systems, where insulin delivery can now be automated by sensor glucose-driven algorithms (6–9).

The move from patient/healthcare provider-based control of glycemia to algorithm (device) driven therapy is associated with major psychosocial changes. A study with patients from the United Kingdom observed that Hybrid Close Loop (HCL) systems at three months improved glucose control, diabetes management, and quality of life measures such as fear and worry of hypoglycemia in young patients with type 1 diabetes (T1D) and their carers (10). Similar observations concern the improvement of metabolic control and quality of life after introducing MiniMed 780G™in an Australian population of children and adolescents (11, 12). On the other hand, the results of studies on the impact of the MiniMed 780G™Advanced Hybrid Close Loop (AHCL) system on the quality of sleep in a population of adolescents were inconsistent (13, 14). There are also some observations indicating that the MiniMed 780G™ system may be helpful in patients with T1D and comorbid mental health issues, but this field requires further thorough investigation (15).

Recently we published a randomized control study in which we indicated that a population of patients with T1D previously naïve to advanced technology, who decided to undergo transition from multiple daily injections (MDI) and self-monitoring of blood glucose (SMBG) directly to the MiniMed 780G™ AHCL, experienced a significant improvement in selected aspects of quality of life: feeling well, working, eating as I would like, and doing normal things in as short time as 3 months after the transition. In addition, the patients from the AHCL group experienced lower levels of stress, fewer feelings of guilt, and could more easily be in contact with their emotions in stressful situations (16, 17). It was the first such study investigating psychological parameters in a population that has undergone the most extreme transformation from naïve to technology to advanced hybrid closed-loop therapy.

In the current study, we aimed to examine whether the improvement in quality of life in the same group of patients changed or was sustained after 1 year of MiniMed 780G™use and if there were any other significant changes in the psychological parameters of the examined population.

## Methods

This was a 9-month observational continuation of the previous 3-month randomized controlled trials (RCT) project, in which we compared the results from the beginning of the study (month 0 with those obtained after 12 months altogether) (8). After the first 3 months of the RCT phase, patients from the AHCL arm continued the follow-up for additional 9 months. The only change in treatment concerned the sensor use – at month 6 the patients were switched from Guardian™ Sensor 3 (GS3) to Guardian™ 4 Sensor (GS4) (calibration-free sensor). The glucose control outcomes were reported by us in a separate research paper (6, 17).

The studied population consisted of 20 T1D technology naïve individuals. After the first 3 months, two male participants withdrew from the study: one due to difficulties in following the protocol and the second due to adhesive issues of infusion sets and sensors during work in high-temperature conditions. A total of 18 patients (10 men, 8 women; age 40.9 ± 7.6 years) completed a 9-month follow-up on a MiniMed 780G™ pump.

The patients filled out a set of questionnaires at the beginning, after 3 months (16), and after 12 months of the study. After the 3 months stage, the control group ended, and the patients from the studied group continued for an observational period of 9 months. The patients had one visit every 3 months during the 9-month follow-up and one more additional visit to change the type of the sensor from Guardian GS3 to GS4. They did not have additional contact with the clinical team but they were able to get technical support from the company helpline when needed.

For these analyses, we compared the results from the beginning of the study with those observed after a year of study continuation. The following tests were used and considered in the analysis:

State-trait anxiety inventory: this is a tool that allows us to assess anxiety defined as a situational state (State Anxiety X1) of the patient and anxiety measured as a relatively stable personality trait (Trait Anxiety X2). The state anxiety scale assesses the current state of anxiety, nervousness, worry, and tension in a given moment of activation of the autonomic nervous system. The trait anxiety scale assesses the tendency of the patient to react with apprehension and worry in general, and anxiety measured here is understood as a trait of personality. Each subscale consists of 20 items (16, 18). Response for X1 assesses the intensity of current feelings evaluated by answering 1) not at all, 2) somewhat, 3) moderately so, and 4) very much so. Responses for X2 evaluate the frequency of feelings in general: 1) almost never, 2) sometimes, 3) often, and 4) almost always (18)

Generalized self-efficacy scale: a self-report scale measuring self-efficacy. It reflects patients’ confidence in the ability to exert control over their own motivation, behavior, and social environment. The construct of perceived self-efficacy reflects the belief that one can perform novel or difficult tasks in various domains of functioning. The scale is a self-administered 10-item tool that requires 4 minutes response time on average. Responses are made on a 4-point scale: 1 = Not at all true, 2 = Hardly true, 3 = Moderately true, and 4 = Exactly true. The results are added to a composite score from 10 to 40. Each item refers to efficient and successful coping from the internal perspective (16, 19).

Quality of life in diabetes questionnaire (20): a tool that assesses the QoL of adults with T1DM. Validation of the Polish version, based on the Mapi Research Trust license, included forward translation by a health professional in clinical psychology and psychiatry, an expert panel analysis of the translation, back translation by a native speaker, and a pilot study on a sample of patients with T1DM. The questionnaire is a self-assessment scale composed of two parts. The first part measures the QoL with diabetes in one out of 23 life areas. In the second part, the patient assesses the importance of each of the 23 aspects of life. The mean value of the global QoL is 138 points and the maximum test result is 345 points. The mean value for a given area is 6, while the maximum for a given area is 15. The higher the result, the better the patient’s QoL (16, 21).

To compare two dependent groups, a paired t-test or a non-parametric alternative when appropriate was used. To compare three or more paired groups, an ANOVA or Friedman test was used. Cohen’s d-effect size (ES) was used to assess the magnitude of the experimental effect. All statistical analyses were performed with R, version 4.2.2.

### Ethical considerations

The study was approved by the bioethics committee (no. 1072.61201.8.2020, dated May 28, 2020, trial registry no. NCT04616391). All patients provided written informed consent to participate in this study. The collected data were stored anonymously on an encrypted disc in the hospital according to recommendations from the bioethics committee. The participants did not receive any financial compensation for participation in the study.

## Results

The baseline characteristics of the examined population are presented in **Table 1**.

**Table 1:** Characteristics of the studied population at baseline (N=18; 10 men, 18 women).

Variable	Mean ± SD
Age [years]	40.9 ± 7.8
Diabetes duration [years]	18.7 ± 11.9
HbA1c at enrolment [%]	7.1 ± 0.9
BMI [kg/m^2^]	24.4 ± 3.0

The metabolic outcomes of the patients are available in the research paper by Matejko et al. (17) and are presented below in **Table 2**.

**Table 2:** Metabolic outcomes, 12 months of follow-up.

One-year follow-up period					
Metrics	Quarter 1	Quarter 2	Quarter 3	Quarter 4	Avg ± SD
Sensor glucose outcomes					
Avg SG (mg/dL)	132.3 ± 7.1	133 ± 9.8	137.6 ± 11.8	136.3 ± 10.2	134.8 ± 9.9
SD of SG (mg/dL)	41.2 ± 5.1	41.3 ± 5.5	41.5 ± 7.0	41.4 ± 7.3	41.4 ± 6.2
GMI (%)	6.5 ± 0.2	6.5 ± 0.2	6.6 ± 0.3	6.6 ± 0.2	6.5 ± 0.2
% of time SG <54 mg/dL	0.4 ± 0.4	0.4 ± 0.5	0.2 ± 0.3	0.3 ± 0.3	0.3 ± 0.4
% of time SG <70 mg/dL	2.1 ± 1.4	2.1 ± 1.5	1.4 ± 1.3	1.5 ± 1.2	1.7 ± 1.4
% of time SG 70–180 mg/dL	84.8 ± 4.4	84.1 ± 5.3	83.2 ± 6.5	84.0 ± 6.4	84.0 ± 5.6
%of time SG >180 mg/dL	11.4 ± 3.5	12.0 ± 5.0	13.3 ± 5.7	12.3 ± 5.2	12.3 ± 4.9
% of time SG >250 mg/dL	1.4 ± 1.0	1.5 ± 1.1	1.9 ± 1.7	2.0 ± 1.7	1.7 ± 1.4

SG, sensor glucose; SD, standard deviation; GMI, Glucose Management Indicator; N, number; Avg, average.

Values are presented by Mean ± SD (Median).

The results obtained after 3 months are presented in **Table 3**, while a thorough analysis of the results is presented in separate studies (6, 16).

**Table 3:** Psychological results after a 3-month randomized clinical trial.

Category	3-months randomized clinical trial	
	M	SD
STAI: State-Trait Anxiety Inventory.		
STAI X1 score	32.12	7.84
STAI X1 sten	4.06	2.11
STAI X2 score	37.35	8.59
STAI X2 sten	4.18	2.7
GSES: Generalized Self-Efficacy Scale.		
GSES score	32	4.31
GSES sten	7.31	1.45
QoL-Q Diabetes		
Global QoL score	201.28	55.49
QoL family relationships/friendships	10	3.66
QoL going out or socializing	8.38	2.78
QoL partner/spouse relationship	9.67	4.16
QoL enjoying sexual activity	8.83	3.2
QoL being physically active	8.89	2.83
QoL feeling well^g^	9.83	3.2
QoL feeling in control of my body	8.83	3.38
QoL looking good	7.67	2.59
QoL having holidays	9.22	3.54
QoL working^g^	10.41	3
QoL affording the things I would like	9	3.66
QoL driving	9.06	3.28
QoL practicing my religion	8.27	3.95
QoL sleeping	9.47	3.08
QoL eating as I would like	7	3.41
QoL looking after or being useful to others	8.65	1.97
QoL pets/animals	8.75	3.79
QoL being independent	10.82	3.05
QoL being in control of my life	10.29	3.46
QoL being spontaneous	8.47	4.54
QoL doing a “normal” thing	9.88	3.82
QoL being treated as “normal”	9.29	4.25
QoL having confidence	9.35	3.37

The results obtained with the use of psychological tests are presented in **Table 4**.

**Table 4:** Outcomes of anxiety, self-efficacy, and quality of life – comparison of results at the beginning and at the end of the study, 12-month follow-up.

Category	Beginning of the study		End of the study (12 months)		Cohen’s d effect size	Absolute difference in mean scores	P value
	M	SD	M	SD			
STAI: State-Trait Anxiety Inventory.							
STAI X1 score	37.5	8.48	33.06	10.25	0.46	4.44	.08
STAI X1 sten	5.12	1.93	3.88	2.53	0.56		**.04**
STAI X2 score	40.44	8.87	36.56	10.27	0.60	3.88	**.02**
STAI X2 sten	4.83	2.62	3.83	2.87	0.60		**.02**
GSES: Generalized Self-Efficacy Scale.							
GSES score	30.4	2.17	31.8	3.16	0.76	1.4	**.03**
GSES sten	6.9	0.88	7.1	0.99	0.32		.42
QoL-Q Diabetes							
Global QoL score	185.41	32.03	209.94	39.73	0.61	24.53	**.02**
QoL family relationships/friendships	9.65	3.24	10.94	2.3	0.35	1.29	.17
QoL going out or socializing	8.5	2.34	10.06	2.08	0.50	1.56	.65
QoL partner/spouse relationship	9.82	3.11	10.76	3.31	0.32	0.94	.20
QoL enjoying sexual activity	8.25	2.38	9.81	3.58	0.46	1.56	.08
QoL being physically active	7.88	2.15	11.06	3.63	0.71	3.72	**.02**
QoL feeling well^g^	7.71	3.14	10.94	2.95	0.73	2.93	**<.01**
QoL feeling in control of my body	7.71	2.39	9.88	3	0.72	2.17	**<.01**
QoL looking good	7.29	2.2	9.47	2.72	1.07	2.18	<.01
QoL having holidays	9.12	3.52	9.94	3.56	0.23	0.82	.36
QoL working^g^	7.29	1.99	9.76	2.56	1.12	2.47	<.01
QoL affording the things I would like	8.65	2.8	9.06	2.82	0.13	0.41	.59
QoL driving	8.53	3.45	8.82	3.7	0.08	0.29	.75
QoL practicing my religion	8.47	4.07	9	4.41	0.16	0.53	.82
QoL sleeping	8.62	2.6	10.31	3.38	0.66	1.69	.01
QoL eating as I would like	5.06	2.62	8	3.97	0.79	2.94	**<.01**
QoL looking after or being useful to others	9	2.28	10.69	2.41	0.65	1.69	.02
QoL pets/animals	7.69	2.02	10.15	3.18	0.95	2.46	<.01
QoL being independent	9.44	3.1	10.62	2.68	0.32	1.18	0.22
QoL being in control of my life	8.94	3.02	10.12	2.36	0.39	1.18	.14
QoL being spontaneous	6.27	2.74	8.67	3.42	0.67	2.4	**.02**
QoL doing a “normal” thing	7.47	3	10.13	3.2	0.67	2.66	**.02**
QoL being treated as “normal”	9.31	2.91	9.88	3.18	0.21	0.57	.41
QoL having confidence	8.88	2.16	10	2.88	0.59	1.12	.05

P<0.05. Bold values mean statistically significant p-value.

As indicated in **Table 4**, a statistically significant increase in QoL was reported in the global score (p=0.02) and in as many as 11 out of 23 analyzed areas of life: being physically active (p=0.02), feeling well (p<.01), feeling in control of my body (p<.01), looking good (p<.01), working (p<.01), sleeping (p<.01), eating as I would like (p<01), looking after or being useful to others (p= 0.02), being active with pets/animals (p<01), being spontaneous (p=0.02), and doing “normal” things (p=0.02). Both state (p=0.04) and trait (p=0.02) anxiety decreased while general self-efficacy significantly increased (p=0.03).

There were no domains where a reduction in quality of life was apparent.

## Discussion

To the best of our knowledge (17), this is the first long-term follow-up study investigating the psychological well-being of adult people with T1D previously naïve to diabetes technology (treated with MDI and SMBG) who experience a direct switch to the AHCL system with novel calibration-free sensors, and the sustainability of the obtained changes in their quality of life. The aim was to evaluate the effect of a one-year use of an advanced hybrid closed-loop system on the quality of life, level of anxiety, and level of self-efficacy in adults previously treated with multiple daily injections and naïve to advanced diabetes technology. The main focus was the adjustment to the new technology not to the glucose levels, although it was also an important factor connected with the new technology use.

Evaluation of patients’ QoL is a well-grounded indicator of the effectiveness of provided healthcare and may be helpful for health professionals and healthcare policymakers in their efforts to improve the well-being of patients (22). In the research paper presenting results after 3 months of transition (16), we reported that the patients experienced a significant increase in four aspects of QoL: feeling well, working, eating as I would like, and doing normal things. This was a substantial change that could be directly associated with a greater level of freedom and safety connected with the change in treatment. However, it became crucial to investigate if changes were not only the result of the initial excitement of the patients, and if the changes could be sustained over time and expanded into other, more specific, aspects of the life of the examined patients. Thus, the 9-month follow-up observation was carried out.

We found that after a year of the study not only were the previously indicated four life areas sustained as much more satisfactory than before the study, but the patients evaluated their quality of life as significantly better in another seven areas: being physically active, feeling in control of my body, looking good, sleeping, looking after or being useful to others, being active with pets/animals, and being spontaneous. An in-depth exploration of those aspects shows that, with time, the patients adjusted to the MiniMed 780G™ pump and more willingly started to experience various life activities, including social relations.

Although there were no radical changes in the food choices of the examined patients, they experienced a greater feeling of freedom in terms of eating. The higher level of freedom in physical activity could be explained by the fact that patients on a hybrid closed loop achieve better glycemic control during their everyday activities (23) and thus they are more willing to undertake various spontaneous activities, such as playing with pets. Patients claimed to feel much more spontaneous than during previous treatment methods. Also worthy of note is that, although the patients started to wear a tethered personal insulin pump (with connecting tubes) and sensors on their body, their subjective assessment of their attractiveness also increased, which can be connected with their general better functioning (24). It is worth mentioning in this respect that even issues of intimacy assessed by asking QoL with regard to enjoying sexual activity showed positive trends (p=0.08).

The observed improvement in sleep quality is consistent with a study on children and young adults with T1D (and their parents) on 780G, but this observation was from a shorter period of time (25, 26). The possible factors contributing to the improvement in the quality of sleep could be fewer hypoglycemia episodes, lower glucose variability, and no need for calibration since the G4S introduction in the course of our follow-up study.

The improvement in the quality of life of the patients undergoing the transition increased not only in the 11 subscales measured but also in the global score of the QoL. This suggests that the patients were not only able to obtain better functioning in selected aspects of their life with diabetes but also that after the 12 months of the study, they achieved generally better life satisfaction, self-esteem, well-being, and meaning of life (2). The improvement in the global indicator of QoL observed in our study can be associated with many component factors found also in other studies (11, 12), such as less exhaustion, more energy, less stress while on AHCL, less thinking about the disease, and better diabetes management, and improved diabetes treatment satisfaction may be a possible consequence of reduced worry and increased trust in AHCL (11). These factors might also result in a decrease in diabetes-related emotional distress and, thus, improvement of QoL (12).

When analyzing the possible sources of such essential change, attention should be paid to the issue of anxiety. Patients with diabetes often struggle with the fear of hypoglycemia (20, 27). One of the ways patients with diabetes deal with this fear is to sustain glucose at slightly elevated levels. This may evoke a fear of complications, potentially creating a cycle of anxiety (28). Our patients undergoing the transition in treatment after the year of follow-up experienced a significant decrease in both state and trait anxiety. We suggest that, to a high extent, this could be associated with much lower glucose variability, lower time spent below range (<70 mg/dL and 54 mg/dl), and a greater general metabolic safety of the patients. This, in turn, could potentially be one of the major factors resulting in the increase in QoL.

There was a significant increase in the self-efficacy of the patients. Self‐efficacy is described as a cognitive process where, through environmental and social influence, individuals learn new behaviors that affect their ability to improve future events (29). Enhancing self‐efficacy can improve the clinical outcomes and quality of life for patients living with chronic diseases (29). Self-efficacy is an important factor in the management of self-care among young adults with T1D; which, in turn, can be an important mechanism by which self-efficacy influences HbA1c levels (30). In our analyses, after 12 months of 780 G use, our patients displayed much better self-efficacy. It could be assumed that their better metabolic control gave them a sustained feeling of being able to “grab the disease in required limits” as stated by one of the patients. One can speculate that the treatment diverted cognitive potential from managing glucose and fear of hypoglycemia to expanding patients’ capabilities and potentials. 

During the study, a new calibration-free GS4 sensor during follow-up was introduced which could be one of the additional factors that contributed to the QoL increase (6), especially in terms of quality of sleep. The patients were not woken up by the need for night calibrations, as it was with S3G, and also they did not have to wake up during the night to check their blood glucose with glucometers, as they did before the 780 G pump usage.

The study has some limitations. One of them is the number of participants and we consider it essential to carry out similar investigations on a greater population. Additionally, the patients had the possibility to discuss their psychological well-being with a clinical psychologist throughout the whole period of the study. Throughout the whole study, only three of the patients asked for such a consultation and they were minor ones, not connected directly with the MiniMed 780G™ pump, but the very fact that the patients felt safe because of such a possibility could have some moderating effect on the quality of life. Another limitation is the lack of continuation of the control group; we observed them only during the 3 months of the initial stage as agreed in the protocol, and later on, the observation included only the studied group on the 780G pump. One of the reasons was the ethical aspect – we did not want to block the patients from the control group from their use of modern technologies for as long as 9 additional months.

Some limitations may also arise from the fact that we did not evaluate the quality of life after the switch from sensor S3G to S4G. However, we could not predict at which moment the impact of the switch could be the isolated factor having an impact on the very complex psychological parameters. We assumed that this was rather an additional factor playing a role in the observed improvement, important especially in terms of the quality of sleep, which we touched upon in the discussion. To assume that this one specific factor was so essential for the whole adaptation to the system could be misleading.

In addition, we did not perform individual profile analyses - in some QoL areas the results may be not uniform over patients, and patients’ experiences may diverge over time. However, in this study, we wanted only to show a general trend of change for the whole study group.

Nevertheless, the obtained results show that the transition directly from MDI and SMBG to the MiniMed 780G™ system resulted in substantial growth in quality of life, sustained over time, much better self-efficacy, and a lower level of anxiety. This, combined with the great improvement in metabolic control, can be considered comprehensive progress in the treatment of patients with type one diabetes.

## Data availability statement

The raw data supporting the conclusions of this article will be made available by the authors, without undue reservation.

## Ethics statement

The studies involving humans were approved by the bioethics committee of Jagiellonian University Medical College (no. 1072.61201.8.2020, date May 28, 2020, trial registry no. NCT04616391). The studies were conducted in accordance with the local legislation and institutional requirements. The participants provided their written informed consent to participate in this study.

## Author contributions

KC: writing of the paper, selecting the research tools, analyzing the results, participating in the study on each of its stage as a research team member, BM: co-writer of the paper, statistical analyses, leader of the research team, AJ: editing of the text, member of the research team, BK-W: member of the research team, OC: member of the research team, analyzing the results, editing the text, MM: editing the text, participating in creating of the discussion, language correction, TK: senior author, the main researcher and originator of the study. All authors contributed to the article and approved the submitted version.

## References

[1] Haraldstad K Wahl A Andenæs R Andersen JR Andersen MH Beisland E. A systematic review of quality of life research in medicine and health sciences. Qual Life Res (2019) 28(10):2641–50. doi: 10.1007/s11136-019-02214-9 PMC6761255 31187410

[2] Oluchi SE Manaf RA Ismail S Kadir Shahar H Mahmud A Udeani TK. Health related quality of life measurements for diabetes: A systematic review. Int J Environ Res Public Health (2021) 18(17):9245. doi: 10.3390/ijerph18179245 34501838 PMC8431362

[3] Rodríguez-Almagro J García-Manzanares Á Lucendo AJ Hernández-Martínez A. Health-related quality of life in diabetes mellitus and its social, demographic and clinical determinants: A nationwide cross-sectional survey. J Clin Nurs (2018) 27(21-22):4212–23. doi: 10.1111/jocn.14624 29987853

[4] Gálvez Galán I Cáceres León MC Guerrero-Martín J López Jurado CF Durán-Gómez N. Health-related quality of life in diabetes mellitus patients in primary health care. Enferm Clin (Engl Ed) (2021) 31(5):313–22. doi: 10.1016/j.enfcle.2021.03.003 34376354

[5] Sayyed Kassem L Aron DC. The assessment and management of quality of life of older adults with diabetes mellitus. Expert Rev Endocrinol Metab (2020) 15(2):71–81. doi: 10.1080/17446651.2020.1737520 32176560

[6] Matejko B Juza A Kieć-Wilk B Cyranka K Krzyżowska S Cohen O. One-year follow-up of advance hybrid closed-loop system in adults with type 1 diabetes previously naive to diabetes technology: The effect of switching to a calibration-free sensor. Diabetes Technol Ther (2023) 25(8):554–8. doi: 10.1089/dia.2023.0059 37184526

[7] Foster NC Beck RW Miller KM Clements MA Rickels MR DiMeglio LA. State of type 1 diabetes management and outcomes from the T1D exchange in 2016–2018. Diabetes Technol Therap (2019) 21:66–72. doi: 10.1089/dia.2018.0384 30657336 PMC7061293

[8] Schiaffini R Deodati A Nicoletti MC Carducci C Ciampalini P Lorubbio A. Comparison of two advanced hybrid closed loop in a pediatric population with type 1 diabetes: a real-life observational study. Acta Diabetol (2022) 59(7):959–64. doi: 10.1007/s00592-022-01886-z 35451679

[9] Petrovski G Al Khalaf F Campbell J Umer F Almajaly D Hamdan M. One-year experience of hybrid closed-loop system in children and adolescents with type 1 diabetes previously treated with multiple daily injections: drivers to successful outcomes. Acta Diabetol (2021) 58(2):207–13. doi: 10.1007/s00592-020-01607-4 PMC7548407 33044604

[10] Ng SM Katkat N Day H Hubbard R Quinn M Finnigan L. Real-world prospective observational single-centre study: Hybrid closed loop improves HbA1c, time-in-range and quality of life for children, young people and their carers. Diabetes Med (2022) 39(7):e14863. doi: 10.1111/dme.14863 35488481

[11] Abraham MB de Bock M Smith GJ Dart J Fairchild JM King BR. Effect of a hybrid closed-loop system on glycemic and psychosocial outcomes in children and adolescents with type 1 diabetes: A randomized clinical trial. JAMA Pediatr (2021) 175(12):1227–35. doi: 10.1001/jamapediatrics.2021.3965 PMC8506294 34633418

[12] Gianini A Suklan J Skela-Savič B Klemencic S Battelino T Dovc K. Patient reported outcome measures in children and adolescents with type 1 diabetes using advanced hybrid closed loop insulin delivery. Front Endocrinol (Lausanne) (2022) 13:967725. doi: 10.3389/fendo.2022.967725 36060958 PMC9437950

[13] Cobry EC Hamburger E Jaser SS. Impact of the hybrid closed-loop system on sleep and quality of life in youth with type 1 diabetes and their parents. Diabetes Technol Ther (2020) 22(11):794–800. doi: 10.1089/dia.2020.0057 32212971 PMC7698988

[14] Wheeler BJ Collyns OJ Meier RA Betts ZL Frampton C Frewen CM. Improved technology satisfaction and sleep quality with Medtronic MiniMed® Advanced Hybrid Closed-Loop delivery compared to predictive low glucose suspend in people with Type 1 Diabetes in a randomized crossover trial. Acta Diabetol (2022) 59(1):31–7. doi: 10.1007/s00592-021-01789-5 34453208

[15] Tekielak A Seget S Rusak E Jarosz-Chobot P. Can the AHCL system be used in T1D patients with borderline TDDI? A case report. Sensors (Basel) (2021) 21(21):7195. doi: 10.3390/s21217195 34770502 PMC8587306

[16] Cyranka K Matejko B Juza A Kieć-Wilk B Krzyżowska S Cohen O. Improvement of selected psychological parameters and quality of life of patients with type 1 diabetes mellitus undergoing transition from multiple daily injections and self-monitoring of blood glucose directly to the miniMed 780G advanced hybrid closed-loop system: *Post hoc* analysis of a randomized control study. JMIR Form Res (2023) 7:e43535. doi: 10.2196/43535 36692945 PMC9906310

[17] Matejko B Juza A Kieć-Wilk B Cyranka K Krzyżowska S Chen X. Transitioning of people with type 1 diabetes from multiple daily injections and self-monitoring of blood glucose directly to miniMed 780G advanced hybrid closed-loop system: A two-center, randomized, controlled study. Diabetes Care (2022) 45(11):2628–35. doi: 10.2337/dc22-0470 PMC9862281 35972259

[18] Julian LJ. Measures of anxiety: state-trait anxiety inventory (STAI), beck anxiety inventory (BAI), and hospital anxiety and depression scale-anxiety (HADS-A). Arthritis Care Res (Hoboken) (2011) 63 Suppl 11(0 11):S467–72. doi: 10.1002/acr.20561 PMC3879951 22588767

[19] Schwarzer R Jerusalem M. Generalized Self-Efficacy Scale. In: Weinman J Wright S Johnston M, editors. Measures in Health Psychology: A User's Portfolio. Causal and Control Beliefs. Windsor, UK: NFER-Nelson (1995).

[20] Fidler C Elmelund Christensen T Gillard S. Hypoglycemia: an overview of fear of hypoglycemia, quality-of-life, and impact on costs. J Med Econ (2011) 14(5):646–55. doi: 10.3111/13696998.2011.610852 21854191

[21] Speight J Woodcock AJ Reaney MD Amiel SA Johnson P Parrott N. (2010). The QoL-Q diabetes' : a novel instrument to assess quality of life for adults with Type 1 diabetes undergoing complex interventions including transplantation. Diabetic Medicine. (2010) 27(Supplement 1):3–4. Available at: https://hdl.handle.net/10536/DRO/DU:30036424.

[22] Kontoteza IV Dragamestianou A Galanis P Siskou O Papazafiropoulou A Konstantakopoulou O. Investigating diabetes mellittus impact on various aspects of patients' Quality of life. Stud Health Technol Inform (2022) 295:470–3. doi: 10.3233/SHTI220767 35773913

[23] Eckstein ML Weilguni B Tauschmann M Zimmer RT Aziz F Sourij H. Time in range for closed-loop systems versus standard of care during physical exercise in people with type 1 diabetes: a systematic review and meta-analysis. J Clin Med (2021) 10(11):2445. doi: 10.3390/jcm10112445 34072900 PMC8198013

[24] Harrington JM. Implications of treatment on body image and quality of life. Semin Oncol Nurs (2011) 27(4):290–9. doi: 10.1016/j.soncn.2011.07.007 22018408

[25] Beato-Vibora PI Gallego-Gamero F Lazaro-Martin L Romero-Perez MDM Arroyo-Diez FJ. Prospective analysis of the impact of commercialized hybrid closed-loop system on glycemic control, glycemic variability, and patient-related outcomes in children and adults: a focus on superiority over predictive low-glucose suspend technology. Diabetes Technol Ther (2020), 22 (12)912–9. doi: 10.1089/dia.2019.0400 31855446

[26] Ng SM Wright NP Yardley D Campbell F Randell T Trevelyan N. Real world use of hybrid-closed loop in children and young people with type 1 diabetes mellitus-a National Health Service pilot initiative in England. Diabetes Med (2023) 40(2):e15015. doi: 10.1111/dme.15015 36424877

[27] Bloomgarden Z. Fear of hypoglycemia. J Diabetes (2017) 9(2):108–10. doi: 10.1111/1753-0407.12491 27736014

[28] Buchberger B Huppertz H Krabbe L Lux B Mattivi JT Siafarikas A. Symptoms of depression and anxiety in youth with type 1 diabetes: A systematic review and meta-analysis. Psychoneuroendocrinology. (2016) 70:70–84. doi: 10.1016/j.psyneuen.2016.04.019 27179232

[29] Farley H. Promoting self-efficacy in patients with chronic disease beyond traditional education: A literature review. Nurs Open (2019) 7(1):30–41. doi: 10.1002/nop2.382 31871689 PMC6917929

[30] Johnston-Brooks CH Lewis MA Garg S. Self-efficacy impacts self-care and HbA1c in young adults with Type I diabetes. Psychosom Med (2002) 64(1):43–51. doi: 10.1097/00006842-200201000-00007 11818585

